# Presence of anti-mitochondrial antibodies and elevated serum immunoglobulin G levels: is this primary biliary cirrhosis-autoimmune hepatitis overlap syndrome?

**DOI:** 10.17179/excli2014-660

**Published:** 2015-01-30

**Authors:** Najihan Abdul Samat Muttaqillah, Asrul Abdul Wahab, Chuan Hun Ding, Marlyn Mohammad, Suvra Biswas, Md. Mostafizur Rahman

**Affiliations:** 1 Department of Medical Microbiology & Immunology, Universiti Kebangsaan Malaysia Medical Centre (UKMMC), 56000 Cheras, Kuala Lumpur, Malaysia

**Keywords:** autoimmune hepatitis, anti-mitochondrial antibody, anti-nuclear antibody, overlap syndrome, primary biliary cirrhosis

## Abstract

Primary biliary cirrhosis in combination with autoimmune hepatitis has been termed “overlap syndrome”, but its diagnosis is challenging. We report a case of a 43-year-old lady who presented with a six-month history of jaundice and pruritus. She subsequently developed gum bleeds. Laboratory investigations revealed hypochromic microcytic anemia, abnormal coagulation profiles, elevated serum alanine transferase and alkaline phosphatase levels, and raised serum IgG and IgM levels. Her serum was also positive for anti-nuclear and anti-mitochondrial antibodies. The findings from her abdominal CT scan were suggestive of early liver cirrhosis and the histopathological examination results of her liver biopsy were consistent with primary biliary cirrhosis. The patient was treated with ursodeoxycholic acid and her liver function test parameters normalized after six months.

## Introduction

Autoimmune hepatitis (AIH), primary biliary cirrhosis (PBC) and primary sclerosing cholangitis (PSC) are autoimmune liver diseases. These conditions can be differentiated based on clinical, biochemical, immunological and histological features. They are classically viewed as distinct entities although shared patterns across the spectrum have been recognized (Silveira, 2013[10]). Primary biliary cirrhosis (PBC) is a chronically progressive cholestatic disease of the liver. It is characterized by immune-mediated destruction of small intra-hepatic bile ducts, causing cholestasis initially and later cirrhosis. The autoimmune basis of PBC is supported by the presence of specific anti-mitochondrial antibodies (AMA) and auto-reactive T cells (Bowlus and Gershwin, 2014[2]). Autoimmune hepatitis (AIH) is a progressive inflammatory liver disorder characterized by inflammatory liver histology, circulating non-organ-specific autoantibodies, and increased levels of immunoglobulin G but with an unknown etiology (Mieli-Vergani et al., 2009[6]). 

## Case report

A 43-year-old Malay housewife with no previous medical history noticed yellowish discoloration of her sclera and skin associated with generalized pruritis for six months. She initially visited the general practitioner, who screened her for viral hepatitis but she was negative for hepatitis B and C. Subsequently, she developed gum bleeds and was investigated in UKM Medical Centre where she also received a blood transfusion due to low hemoglobin (4 g/dL). She was asymptomatic for anemia and had no symptoms of biliary obstruction. She does not consume alcohol or traditional remedies. On examination, she was jaundiced and pale. She had clubbed fingers, but there were no stigmata of chronic liver disease. Abdominal examination revealed hepatomegaly. Her relevant blood investigation results are shown in Table 1(Tab. 1).

A full blood picture (FBP) examination showed microcytic hypochromic erythrocytes with anisopoikilocytosis, which was compatible with iron deficiency anaemia. Her alpha-fetoprotein level was normal. An abdominal CT scan showed ill-defined hypodensities in the liver, suggesting regenerating nodules of early liver cirrhosis. Further immunological investigations showed raised serum immunoglobulins M and G levels (355 and 2870 mg/dL respectively), but the immunoglobulin A level was normal. Serum anti-nuclear antibodies (ANA) were detected and formed a speckled pattern on HEp-2 cells. Anti-mitochondrial antibodies were also positive up to a titre of 1:80. Anti-double stranded DNA and anti-smooth muscle antibodies were not detected. The provisional diagnosis of primary biliary cirrhosis with possible autoimmune hepatitis was made. 

Four months later, she presented again with severe anemia (hemoglobin of 2.5 g/dL) secondary to menorrhagia and coagulopathy. She was transfused with six units of packed cells and four units of fresh frozen plasma. Further investigations of her anemia were undertaken. A bone marrow examination revealed erythroid hyperplasia, in keeping with iron deficiency. Thalassemia was ruled out by haemoglobin analysis. A liver biopsy showed mild to moderate chronic inflammatory portal tract infiltrates consisting of lymphocytes, polymorphs and plasma cells. These cells expanded and partially obliterated the tracts. There was also significant portal fibroplasia, bile ductular proliferation, portal-to-portal reticulin collapse and fibrosis. Intra-cytoplasmic and canalicular cholestasis were noted. Lobular inflammation was present but with no hepatocellular necrosis.

These features were consistent with active primary biliary cirrhosis and the patient was treated with ursodeoxycholic acid (UDCA) (750 mg od; 15 mg/kg/day). Ferrous fumarate (200 mg tds) was also given for the anemia. Her liver function test parameters improved a month post discharge from our hospital and they normalized during a follow-up at six months.

## Discussion

Despite the absence of standard diagnostic criteria and the lack of agreement on what constitutes an overlap syndrome, PBC with AIH overlaps are recognised, consisting of conditions exhibiting features of both entities. The prevalence varies widely from 1 to 19 percent in patients initially diagnosed with PBC (Silveira, 2013[10]; Heneghan, 2014[4]). Interestingly, the features of autoimmune hepatitis may be present at diagnosis or during follow-up, with clinical recognition being somewhat easier in the latter. Alternately, PBC may also develop in patients who present with AIH (Heneghan, 2014[4]). Although overlap syndrome has been considered a clinical entity characterized by the occurrence of both conditions simultaneously, the transition of one condition to the other has also been reported, and AIH superimposed upon PBC may lead to a rapid progression to cirrhosis and liver failure (Poupon et al., 2006[8]). 

The PBC diagnostic criteria include serum ALP levels raised at least two times the upper limit of normal or serum gamma-glutamyl transpeptidase levels raised at least five times the upper limit of normal, a positive test for AMA, and a liver biopsy showing florid duct lesions (Silveira, 2013[10]). The AIH diagnostic criteria include raised serum ALT levels at least five times the upper limit of normal, raised serum IgG levels at least two times the upper limit of normal or the presence of anti-smooth muscle antibody, and a liver biopsy showing moderate to severe periportal or periseptal lymphocytic piecemeal necrosis (Silveira, 2013[10]). Our patient fulfilled one AIH criterion and all of the PBC criteria. The diagnosis of PBC-AIH overlap syndrome is confirmed when at least two out of three of both criteria are met either simultaneously or consecutively (Silveira, 2013[10]). 

The presence of ANAs in a patient with autoimmune liver disease can be divided into several categories but ANA patterns such as “rim-like” and “multinuclear dots” are likely to be associated with PBC (Romero-Gómez et al., 2004[9]). In AIH, the ANA pattern seen is usually homogeneous (Mieli-Vergani and Vergani, 2011[7]). In this patient, her ANA had a speckled pattern, which is most likely PBC-related. Liver histology plays an important role in diagnosis. Pathological changes in the portal tracts signify PBC, and lobular inflammation has been taken as a feature suggesting AIH in one study (Joshi et al., 2002[5]). 

The International Autoimmune Hepatitis Group (IAIHG) in 2011 issued a consensus statement noting that patients with primary biliary cirrhosis with features of autoimmune hepatitis are to be considered for immunosuppressive treatment (Boberg et al., 2011[1]). The therapeutic goal is to attain normal serum aminotransferase levels and histological improvement. Combination treatment of glucocorticoids and UDCA is effective in improving liver histology within seven years of follow-up in patients conforming to a strictly defined PBC-AIH overlap syndrome, as opposed to UDCA or corticosteroids alone (Chazouillères et al., 1998[3]). In our patient, an improvement in her liver function test was documented during follow-up, despite not being treated with corticosteroids.

In conclusion, the diagnosis of overlap syndrome is challenging but it is imperative to diagnose due to its rapid progression to cirrhosis and liver failure. Patients with laboratory results diagnostic of PBC coupled with findings suggestive of AIH should be carefully followed-up. They may be treated with ursodeoxycholic acid initially but be considered for a trial of corticosteroids if no improvement in the liver function is observed during follow-up. 

## Acknowledgement

The authors express their sincere gratitute to the Dean, Faculty of Medicine, Universiti Kebangsaan Malaysia for his motivation and permission to publish this case report.

## Figures and Tables

**Table 1:**
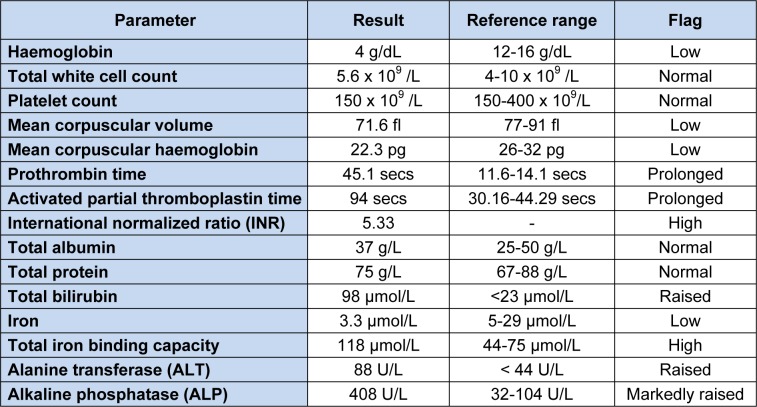
Relevant blood investigation parameters and their results
